# Pharmacists’ Attitudes towards Medically Assisted Dying

**DOI:** 10.3390/pharmacy12020040

**Published:** 2024-02-20

**Authors:** Lun Shen Wong, Shane L. Scahill, Emma Barton, Bert Van der Werf, Jessica Boey, Sanyogita (Sanya) Ram

**Affiliations:** 1 School of Pharmacy, The University of Auckland, 85 Park Road, Grafton, Auckland 1023, New Zealand; 2 School of Epidemiology and Biostatistics, The University of Auckland, 28 Park Ave., Grafton, Auckland 1023, New Zealand

**Keywords:** contemporary challenges, pharmacy practice, professional ethics, implicit bias, expanded roles in pharmacy, conscientious objection in pharmacy

## Abstract

Aims: We aimed to explore pharmacists’ attitudes and support toward medically assisted dying (MaiD) through the End of Life Choice Act 2019 (EOLC), their willingness to provide services in this area of practice, and the influences on their decisions. Methods: The study was conducted via an anonymous, online Qualtrics^TM^ survey of pharmacists. Registered New Zealand pharmacists who agreed to receive surveys from the two Schools of Pharmacy as part of their Annual Practicing Certificate renewal were invited to participate through an email with a Qualtrics URL link. The survey contained questions regarding demographics, awareness, knowledge, support for, and attitudes and willingness to participate. Results: Of the 335 responses received, 289 were valid and included in the analysis. Most participants supported legally assisted medical dying (58%), almost a third of participants did not support it (29%), and 13% of respondents were unsure. The five primary considerations that participants perceived to be beneficial included support from legislation, respect for patient autonomy, discussions around morality, ending suffering, and preserving dignity. The main concerns were legal, personal bias, palliation, stigmatisation, and vulnerability. Conclusions: The influences on the decision by pharmacists to support and willingness to participate in the provision of services consistent with the EOLC are complex and multifactorial. Diverse factors may influence attitudes, of which religion is the most significant factor in not supporting the Act or willingness to participate. Clarity and standardised guidance to ensure that assisted dying queries are appropriately managed in practice would help to address any potential access issues.

## 1. Introduction

Pharmacists are expected to play an integral role in optimising medication therapy, improving health literacy and access to medication to improve health outcomes. Pharmacists can play a vital role in improving quality of life at the end of a patient’s life. Pharmacists’ roles have extended to being involved in the provision of medically assisted end-of-life care in a number of jurisdictions, including the Netherlands, Canada, and Australia [1,2,3].

In New Zealand, the End of Life Choice Act 2019 (EOLC) came into effect on the 1st November 2021. This is the first legislative change of its kind in the New Zealand context, so its application across the health sector, implementation, and analysis of uptake are still in their infancy stages; much is unknown, including the level and expectation of pharmacist participation. The EOLC requires health professionals and all New Zealanders to navigate implementation and reflect changes in societal norms. This legislation gives a person suffering from a terminal illness the ability to lawfully request medical assistance to end their life [4]. In the Act, assisted dying, in relation to a specific person, is defined as “the administration by an attending medical practitioner or an attending nurse practitioner of medication to the person to relieve the person’s suffering by hastening death; or the self-administration by the person of medication to relieve their suffering by hastening death” [4].

Societal norms and the acceptance of medically assisted dying (MaiD) in a multi-cultural society highlight the potential for ethical tensions surrounding conscientious objection (CO) and the rights of healthcare professionals to determine the care they envision they will provide. While officials contend with establishing and implementing the safeguards and processes outlined in the Act, healthcare professionals have also grappled with the practicalities of implementing the law within their realm of practice and navigating the intricacies of balancing CO with reasonable limits, both individually and as a profession [5,6].

The Act ensures that any practitioner who is authorised or needed to do anything under the requirements can conscientiously object and is not required to provide the service [7]. However, they must inform the person being assisted of their objection and refer them to an alternative practitioner who can provide the service in a timely manner. Personal autonomy, private conscience, morals, and personal values are not usurped, as individuals still take responsibility for their decisions. This decision-making process should be supported by their profession’s frameworks, professional standards, and accepted norms [7]. Pharmacists, like other healthcare professionals, are not devoid of personal conscience [8] and may opt out of providing end-of-life service. Person-centred care helps provide a way forward in balancing the duality of private conscience and public role expectations, where patients and healthcare providers tolerate each other’s diverse viewpoints [9]. Since the implementation of MaiD in New Zealand, pharmacist engagement with this service has been limited due to legislative boundaries; however, as further implementation occurs and learnings materialise and professional scopes proliferate, this may not remain so.

The professional guidance for navigating CO, particularly in the context of MaiD, varies between countries. The Pharmacy Council of New Zealand (PCNZ) Code of Ethics does not include a definition of CO; however, the expected approach to service provision is outlined under Principle 4 [10]. The Pharmaceutical Society of Australia Code of Ethics defines conscientious objection as “a practitioner’s refusal to engage or provide a service primarily because the action would violate their deeply held moral or ethical value about right or wrong”. Both regulatory bodies expect those with a CO to ensure that patients are able to access the required care [8,11].

A mapping review of the literature, conducted by Woods et al., reported that most of the research exploring pharmacist involvement in MaiD focused on pharmacists’ attitudes, and opinions concerning assisted dying [12]. Few studies have focused on pharmacists’ experience with MaiD or their willingness to participate. This study aims to explore pharmacists’ attitudes towards MaiD, their willingness to provide services in this area of practice, and what the influences on their decisions are.

The aims of the study are to:

1. Investigate NZ pharmacists’ awareness of and influences on their attitudes toward legalising medically assisted dying under the End of Life Choice Act 2021.
2. Investigate pharmacists’ willingness to provide services consistent with the End of Life Choice Act 2021.

## 2. Methods

The study was conducted via an online Qualtrics^TM^ (Sydney, Australia) survey of pharmacists in New Zealand. Registered New Zealand pharmacists working in the pharmacy sector who have agreed to receive surveys from the Schools of Pharmacy as part of their Annual Practicing Certificate renewal were invited to participate through an email with a Qualtrics™ URL link. There were approximately 3000 pharmacists on this list. The survey was also circulated through pharmacy professional leadership organisations from the end of September until November 2021. This was prior to the EOLC Act coming into force. At this time, the role of pharmacists was ambiguous in how the proposed service would translate into practice.

The survey contained five sections. Section A collected participant demographics, Section B focused on awareness of the End of Life Choice Act 2019, Section C explored attitudes towards MaiD and factors that influenced participants’ views. Section D explored willingness to participate in the provision of EOLC services. Section E explored the psychosocial variables that affect the behaviours of pharmacists in relation to facilitating MaiD. Section C was adapted from a questionnaire exploring physicians’ and nurses’ experiences of MaiD [13,14]. These questions were adapted from a survey of nurses using the framework of the Theory of Planned Behaviour (TPB) to explore responses to requests for assisted dying, with permission from the researchers [14]. The survey was adapted for pharmacists and to suit the New Zealand environment and regulations. The survey was reviewed by three pharmacists with knowledge of the Act and piloted amongst eight pharmacists.

This manuscript is focused on sections A to D of the survey by presenting results on attitudes towards, influences, and willingness to participate in MaiD. 

The study was approved by the University of Auckland Human Participants Ethics Committee on 10 September 2021. Reference Number: UAHPEC22554.

Data analysis was undertaken using the IBM Statistical Package for the Social Sciences (IBM SPSS^TM^ version 19) analytical software and R version 4.3.0 [15]. Descriptive statistics were generated to explore frequency data according to participant characteristics [16]. The multinomial answers were analysed with a logistic regression using the R package lme4 [17]. The assumptions behind the regression were checked with the R package DHARMa [18]. Different models were compared using the Akaike Information Criterium (AIC) [19]. Subsequent pairwise comparisons were made using the parameter estimates and covariances of the best-fitting model. To hypothesise causality between the variables, the R package bnlearn was used to conduct a Bayesian network analysis [20].

Participants’ open text comments were reviewed and categorised into themes using an inductive method [21]. The process involved becoming familiar with the data, then producing codes methodically across the dataset, searching for overarching themes, and creating an index of subthemes. These were tabulated and appraised in investigation team meetings to assess consistency across the dataset. Themes were justified by the investigation team via peer debriefing and critique in research team meetings [21].

## 3. Results

Of the surveys disseminated via the pharmacist regulator emailing list (*n* = 3039), 335 responses were received (11% return rate). Of these, 289 responses (9.5% response rate) were valid due to full survey completion and included in the analysis. The distribution of respondents by role and sector included: community pharmacists (*n* = 120, 35.8%), community pharmacy owners (*n* = 62, 18.5%), hospital pharmacists (*n* = 63, 18.8%), and community pharmacy managers (*n* = 27, 8.1%). In 2021, community pharmacists comprised the majority of pharmacists (78%), and hospital pharmacists were the minority (1%) [22]. These are reflected in the results of this study.

Just over half (58%) supported legalising medically assisted dying; almost a third of participants did not support (29%), and 13% of respondents were unsure (see Table 1). Just over half of the participants by gender, 55% (*n* = 109) female and 65.9% male (*n* = 33), supported the EOLC Act.

Around half (51%) reported having “skim read” the Act. Less than one-fifth (16%) stated that they had read the Act in some detail. Participants reported being somewhat knowledgeable (51%) on the Act, with 22% rating their knowledge as knowledgeable or very knowledgeable. Most participants (83%) had not attended any training related to the Act or its service provision; nevertheless, they had read generally about the proposed medically assisted dying services.

Participants first learned of the EOLC Act mostly through the media (*n* = 131, 45.3%) and the promotion of the EOLC referendum (*n* = 100, 34.6%). Less than 10% were notified about the EOLC Act by their professional pharmacy organisations (*n* = 22, 7.60%) or by the internet (*n* = 11, 3.80%). Participants also learned about the EOLC Act through family members (*n* = 4, 1.4%), friends (*n* = 8, 2.8%), or social media (*n* = 1, 0.30%). Some participants learned about the Act through scientific literature (*n* = 2, 0.70%) and through other means (*n* = 10%, 3.5%).

**Figure 1:**
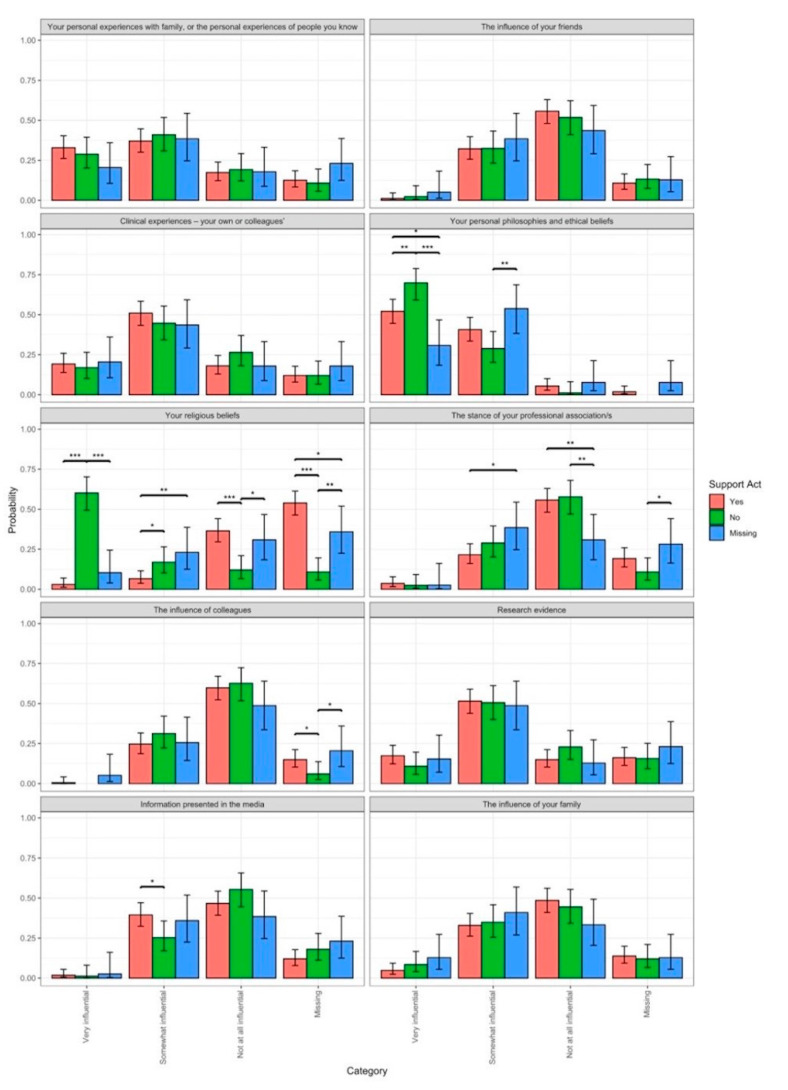
Influences on the decision to support medically assisted dying. [* = *p* < 0.05, ** = *p* < 0.01, and *** = *p* < 0.001].

Participants were asked how influential a list of factors, such as religious beliefs, clinical experiences, personal philosophies and ethical beliefs, the stance of professional organisations, and the influence of colleagues, family, and friends, had been in shaping their views towards medically assisted dying. Figure 1 outlines the perceived influences compared to their support for legalising MaiD.

A multinomial logistic regression was conducted to explore whether the factors of influence differ across levels of support for legalising medically assisted dying (MaiD). There was significant evidence to support differences in mean factors of influence (*p* < 0.05). Post hoc comparisons showed that participants who stated that their personal beliefs and ethical beliefs were very influential were more likely not to support legalising MAiD. There were differences among those participants who found their religious beliefs influential, somewhat influential, or not at all influential. Those who were influenced by religious beliefs did not support legalising MAiD; however, those who were not influenced by religion were supportive of legalising MAiD. There was much missing information or participants who were unsure, and these participants presumably did not identify with being religious and indicated support for legalising MAiD. Those participants who were not influenced by the stance of a pharmacy professional organisation differed from those who were missing information and unsure, presumably due to not belonging to a professional organisation. Those that were somewhat influenced by the media were more likely to support legalising MAiD than those who responded with no to this question.

The decision to not support legalising medically assisted dying was influenced by religious beliefs. A Bayesian network analysis indicated that pharmacists’ willingness to participate in the provision of medically assisted dying consistent with the EOLC was influenced by religious beliefs. The influence of friends, professional leadership organisations, the media, and colleagues was not statistically significant. Pharmacists also noted that organised religion and suicide were emerging concerns relating to legalising medically assisted dying. Pharmacists commented that their religion or beliefs could shape their view of the legislation, and many were not able to separate this bias from their views.

### 3.1. Other Factors Shaping Participants’ Views towards Legalising Medically Assisted Dying

A diverse range of factors were reported to shape participants’ views of EOLC service provision. Themes of influence around service provision include criminality, palliation, vulnerability, suffering and indignity, and autonomy, as shown in Table 2.

Text analysis of pharmacist quotes demonstrated that professional pharmacy organisations and indemnity providers could influence pharmacist views around this service, despite there being no statistically significant difference through pairwise comparison. Participants’ experience as health professionals and the context of the types of patients they worked with could also affect their perception of medically assisted death.

Some respondents noted that culture and access to appropriate services could affect indigenous populations, such as Māori, disproportionately. The role of equity in access to this service is a consideration some pharmacists contemplated when forming their views about legalising end-of-life care.

### 3.2. Main Concerns around Legalising Medically Assisted Dying in New Zealand

Pharmacists presented a range of issues that have been thematically derived into five main concerns (see Table 3) when legalising medically assisted dying in New Zealand. 

### 3.3. Willingness to Provide EOLC-Based Services

Under half of pharmacist participants (*n* = 118, 40.8%) would support providing medically assisted dying consistent with the EOLC Act. Just under one-quarter of pharmacists were undecided (*n* = 71, 24.6%), and just over one-quarter of pharmacists (*n* = 77, 26.6%) would not provide the service. 

Pharmacists that were undecided in their willingness to support EOLC services consistent with that Act tended to have a background in hospital (*n* = 18, 25.4%) or community pharmacy (*n* = 43, 60.6%) practice. They had an average of 11 to 30 years of experience, with a preference noted to be higher in those with 30 and above years of experience within the pharmacy profession. The mean age of this practitioner was either 31–35 years or 46–50 years of age.

Table 4 outlines a thematic analysis of free text responses when asked what services pharmacists would be willing to provide to support the provision of legally assisted dying. Pharmacists reported that they would comply with their scope of practice and provide whatever service they could within that scope. They would engage in obtaining informed consent and referring patients to an appropriate practitioner. Regarding professionalism, pharmacists discussed being comfortable providing counselling and support for patients and their whānau and providing information when requested. Respondents saw themselves as part of a wider multi-disciplinary team and would support increasing awareness of an EOLC service.

Regarding access and services, pharmacists highlighted they would accept providing compounding, dispensing, and medication review services. They were happy to provide a delivery service and procure medicines for this service. Pharmacists also expressed interest in running education sessions and upskilling for this service provision. Table 4 summarises sample quotes from participants regarding possible service provision.

The analysis showed some pharmacists disagreed with taking part in any aspect of this service due to personal or religious beliefs, as demonstrated by the quote below:

“I do not wish to assist on ethical grounds and should have the choice not to do so without repercussion” (Pharmacist 126).

Some participants also reported this service falling outside their scope of practice.

In such cases, some pharmacists noted they would provide a referral service to hospice or palliative care. Some pharmacists also noted with uncertainty that their involvement would depend on other pharmacist peers and professional risk, suggesting that further guidance from a professional pharmacy organisation could be useful in exemplifying the conduct that regulators wish to see. This is consistent with quantitative data whereby those in the “maybe” category (*n* = 21, 74.6%) accounted for a proportion of pharmacists that could support medically assisted dying consistent with the EOLC act, but require further information to make an informed choice about future service engagement.

## 4. Discussion

This study investigated New Zealand pharmacists’ awareness of, support for, and influences on their attitudes toward the End of Life Choice Act and their willingness to provide services consistent with the EOLC Act.

A study from 2019 (*n* = 475) indicated that 67% of nurses (*n* = 318) supported medically assisted dying service provision, whereas only 37% of doctors (*n* = 110) supported medically assisted dying services [23,24]. The public referendum indicated that 65.1% (*n* = 1,893,290) supported the End of Life Choice Act 2019, and 33.7% (*n* = 979,079) indicated that they did not [25]. Just over half (58%) of the pharmacists that responded to the survey supported legalising medically assisted dying; almost a third of participants did not support it (29%), and 13% of respondents were unsure. A small proportion of pharmacists (16%) reported having read the Act in full, and most participants had not attended relevant training courses. This is understandable, as the survey was conducted prior to the Act being implemented. However, it raises the question of the need to provide pharmacists with timely, relevant, and targeted training opportunities. The media played an important role in promulgating information, as 45% of participants first learned of the EOLC Act through the media. 

It is clear that role clarification, education, and training are needed for the pharmacy profession. At the time of the Act passing legislation, there was ambiguity in translating the Act into practice and little guidance on the role of the pharmacist in this process [26,27]. Furthermore, less than one-quarter (22%) of pharmacists reported being knowledgeable or very knowledgeable about the Act. In the absence of media, just over one-third of pharmacists (34.6%) reported learning about the Act through the referendum and 7.6% through a professional pharmacy organisation. The Ministry of Health has acknowledged that pharmacists have important roles in the community setting, and that it is important for pharmacy staff to have an understanding of the law and know where to find information should they be asked about the service [28].

A few participants referred to the emotional distress that could come from handing out assisted dying medication to patients. This is a practice that happens abroad and demonstrates role ambiguity that may have occurred earlier in the implementation phase where the pharmacist role was not clear with regard to what they would or could do within this service provision [1,2].

Moral sensitivity is known to be a key factor in attitudes towards assisted dying. In context, this is the ability of pharmacists to look past their personal belief systems, identify the moral problems associated with assisted dying, and understand the consequences of their decisions as health professionals [29]. There are some pharmacists who would not wish to access assisted dying themselves but feel strongly about people having a choice to end unbearable suffering. There are other pharmacists who believe that assisted dying is wrong, would not access the service themselves, and are unwilling or unable to explore perspectives beyond their own personal belief system. Moral sensitivity appears to be influenced by an individual’s capacity for empathy [30]. Strong themes of empathy toward unbearable suffering emerged in the qualitative data. 

The decision to not support the EOLC Act and not be willing to provide related services was significantly correlated with religious beliefs. Participants who stated their personal and ethical beliefs were very influential were more likely not to support the legalisation of services related to medically assisted dying. 

There was a large proportion of participants who responded with “maybe” or “unsure”. The assumption is made that these participants did not identify with a religion but indicated that they supported the legalisation of medically assisted dying services. Those participants that were unaffected by the attitude of a professional pharmacy organisation differed from those that lacked information in addition to being unsure; presumably because they were not affiliated with a professional pharmacy organisation. Those who indicated that they were somewhat influenced by the media were more likely to support medically assisted dying services than those who answered no to this question. Overall, participants were not significantly influenced by their friends, the stance of professional pharmacy organisations, the media, or colleagues.

Personal belief systems and religion serve as one of the most significant influences on the acceptability of medically assisted dying. This investigation has highlighted the dichotomy that can occur while working as a health professional and holding their own personal beliefs, which drive behaviour. As health professionals, pharmacists are held accountable for fulfilling the minimum competency standards outlined by the New Zealand Pharmacy Council [10]. Humans are highly socialised. Our philosophy of the world around us is largely shaped by our early upbringing, life experiences, cultural or religious norms, and values held by our loved ones or those we spend the most time around [31]. CO clauses are what acknowledge this dichotomy, allowing for compromise when professional obligations tangle with personal belief systems. Religion is the only statistically significant influence on pharmacists’ attitudes toward the assisted dying service in New Zealand. These findings are similar to those of our Australian pharmacist colleagues [1]. Personal belief systems are deeply ingrained in our schema, which highlights the requirement to further explore the perspectives of those undecided pharmacists as a means to improve patient access and experience in this area of healthcare. Being able to recognise one’s conscientious bias with regard to this topic and continuing to provide health services appropriate to patients needs requires further consideration. 

### 4.1. Implications for Policy Makers, Pharmacy Leaders, and Universities

This research highlights the need for tailored continuing education on this topic, aligning with international findings from Australia and Canada [1,4,32]. It raises the important role of professional regulatory bodies in maintaining well-informed health professionals within this evolving healthcare climate. There is a need for guidance documents to be developed and distributed to all pharmacists, and to include how to navigate CO within the community pharmacy context. Continuing education to facilitate the communication skills required for responding to requests, ensuring safe access to the service, and reducing cultural, societal, and existential issues could be considered. Further education around equity and the response of indigenous populations and their views could also be considered [33].

There are a number of pharmacists whose responses suggest the implementation of assisted dying could perpetuate under-investment in downstream palliative care options. This is consistent with concerns about inequity in the quality of palliative care in New Zealand [34]. Additionally, some stated that inequitable access to palliative care could lead to inappropriate uptake of assisted dying. 

### 4.2. Implication of the Findings for Practitioners

A portion of the profession reported they would be better able to participate in assisted dying if there were more precise guidance or codes of conduct from professional pharmacy organisations to allow them to be prepared to handle assisted dying scenarios. Pharmacists commented that there was positive interest in providing dispensing of medications or medication procurement in addition to the delivery of medications, which were traditional aspects of service delivery that pharmacists are familiar with. Pharmacists also noted an interest in participating in continuing education in this area and developing specialty skills or a scope. Nevertheless, clearer direction about definitions of activity within this scope and the provision of such services in a legislatively compliant framework are needed to decrease ambivalence and uncertainty about participation. Furthermore, pharmacists highlighted the need for a protocol for practice to help pharmacists comply with laws surrounding medically assisted dying [1,35].

Whilst the management of CO and the health practitioner’s religion have been issues that have been present in practice, it is important for pharmacies to have systems in place to follow legislative requirements, and to ensure patients can navigate this pathway safely and that professionalism and respect for patient autonomy are upheld [1,35,36,37,38,39,40].

### 4.3. Implications of the Findings for Future Research

The regional locations of participants were not gathered as part of the demographic data. Further investigation should determine whether conscientious-objection-related service and access problems are primarily an urban or regional problem, allowing service providers and health officials to ensure there is adequate geographical overlap and access to practitioners to ensure parity and equity-related access to suitable health services [4]. Further exploration of the one-quarter of undecided pharmacists (*n* = 71, 24.6%) to better understand drivers behind their decision-making regarding service provision could better inform regulatory policy and management around codes of conduct and clarify ambiguity in professional delivery of services. Further investigation into health practitioner personality traits could also be explored to understand the decision-making process with health service provision offerings in comparison to the religion of health practitioners.

## 5. Limitations

Not all pharmacists practicing within New Zealand responded to the survey, making it difficult to generalise with confidence these findings to the wider pharmacist population. The low response rate makes it challenging to arrive at considered decisions regarding pharmacy practice. Non-responder bias was not assessed, and neither was a power calculation. 

It is challenging to ascertain the factors influencing pharmacists’ support or otherwise of the Act. Their perspective has the potential to be influenced by having access to authoritative information about the reality of service provision and how its implementation is proceeding in New Zealand, and further exploration of this could help better inform policy makers on aiding health professionals in decision making in the future. At the time of survey distribution, the Act had not come into effect, so pharmacy organisations and management may not have had time to address concerns raised by pharmacists regarding expectations around service provision. 

Responses may have also been influenced by participants’ assumed likelihood of working in a pharmacy providing the service, or their likelihood of having to converse about assisted dying, rather than the perspective of widening access to end-of-life care to New Zealand society. In this instance, those pharmacists would be required to disclose their CO, and such disclosure can realistically create discontent or discomfort within the workplace. Among these participants, it is likely this has shifted some focus of their responses from access to a health service, and instead, toward their discomfort in working within a health system that has legalised assisted dying.

Overall, the nature of this topic has led to responses being based on personal experience and opinion, rather than factual occurrences in the workplace or the realistic implementation of the service.

## 6. Conclusions

The willingness of pharmacists’ to accept and participate in the process of medically assisted dying is multifactorial. Its nature and complexity have lent themselves to a myriad of attitudes that pharmacists hold towards its implementation. The influences on the decision by pharmacists to support and willingness to participate in the provision of services consistent with the End of Life Choice Act are complex. Diverse factors may influence attitudes, of which religion is the most significant factor in not supporting the Act or willingness to participate in assisted dying. Clarity of process and practice and standardised guidance to ensure that assisted dying queries are appropriately managed in practice would help to address any potential access issues in pharmacy.

Further research exploring how assisted dying has unfolded in New Zealand since the implementation of the End of Life Choice Act is required to help better inform health professionals to make decisions around their involvement in this process.

## Figures and Tables

**Table 1:** Participant demographics and support for legalising medically assisted dying in New Zealand.

Responses to Support for Legalising Medically Assisted Dying in New Zealand								
	Yes		No		Unsure		Total	
**Area of Work**	*n*	%	*n*	%	*n*	%	*n*	%
Community Pharmacy Owner	34	20.4%	15	18.1%	6	16.7%	55	19.2%
Community Pharmacy Manager	16	9.6%	4	4.8%	1	2.8%	21	7.3%
Community Pharmacist	65	38.9%	39	47%	15	41.7%	119	41.6%
Academic Pharmacist	5	3.0%	0	0.0%	1	2.8%	6	2.1%
Hospital Pharmacist	26	15.6%	16	19.3%	8	22.2%	50	17.5%
Other	21	12.7%	9	10.8%	5	13.9%	35	12.2%
	167	100%	83	100%	36	100%	286	100%
Missing response							3	1.0%
**Age**	*n*	%	*n*	%	*n*	%	*n*	%
20–25	18	10.8%	7	8.4%	5	13.9%	30	10.5%
26–30	25	15.0%	11	13.3%	6	16.7%	42	14.7%
31–35	23	13.8%	8	9.6%	5	13.9%	36	12.6%
36–40	22	13.2%	11	13.3%	5	13.9%	38	13.3%
41–45	13	7.8%	7	8.4%	2	5.6%	22	7.7%
46–50	17	10.2%	10	12.0%	3	8.3%	30	10.5%
51–55	20	12.0%	11	13.3%	3	8.3%	34	11.9%
56–60	11	6.6%	7	8.4%	1	2.8%	19	6.6%
61–65	9	5.4%	8	9.6%	5	13.9%	22	7.7%
>65	9	5.4%	3	3.6%	1	2.8%	13	4.5%
	167	100%	83	100%	36	100%	286	100%
Missing response							3	1.0%
**Years of practice**	*n*	%	*n*	%	*n*	%	*n*	%
0–2	18	10.8%	8	9.6%	5	13.9%	31	10.9%
3–5	18	10.8%	11	13.3%	8	22.2%	37	13.0%
6–10	28	16.9%	8	9.6%	3	8.3%	39	13.7%
11–15	22	13.3%	15	18.1%	5	13.9%	42	14.7%
16–20	16	9.6%	5	6.0%	2	5.6%	23	8.1%
21–25	14	8.4%	7	8.4%	3	8.3%	24	8.4%
26–30	16	9.6%	7	8.4%	2	5.6%	25	8.8%
30 and above	34	20.5%	22	26.5%	8	22.2%	64	22.5%
	166	100%	83	100%	36	100%	285	100%
Missing response							4	1.4%

**Table 2:** Factors that influenced participants’ views towards legalising medically assisted dying.

Emerging Theme	Subtheme	Data Quotation Examples
Criminality	Legalities Enforcement Historical Precedence Loopholes and exemptions Interpretation disagreement	“I am not happy with how the law has been written, too many loopholes” (Pharmacist 170) “I have concerns about the end point of this legislation with the right to die extended to an inappropriate extent and with vulnerable people being pressured to make choices they do not actually want” (Pharmacist 40) “I am not against euthanasia, I do believe however that this piece of legislation is very poor” (Pharmacist 241)
Palliation	Service provision Lack of service offer Role of service	“Best practice palliative care should make assisted dying unnecessary” (Pharmacist 115) “I support the principal of assisted dying for individual cases, but, certainly in the UK, and possibly in NZ I would hate future governments to push assisted dying at the expense of good palliative care” (Pharmacist 131) “There has not been an option proposed to increase funding and availability of palliative care to this subset of patients so patients not well managed aren’t provided with the full array of options” (Pharmacist 191)
Vulnerability	Professional ethics Power of attorney Financial burden Disability	“Concern the process may be influenced by people other than the person concerned” (Pharmacist 141) “Politicians start out with an idea but over time the safeguards get whittled away. How long before the end-of-life choice is extended to disabilities or IHC people”. (Pharmacist 148) “Wavering support as this is a personal decision for the person and so it is important to have safeguards—and I’m not sure the Act addresses the people who would most ‘benefit, i.e., the very end of life. It is very situation dependent and I’m unclear how a legal Act can cover all personal situations” (Pharmacist 229)
Suffering and Indignity	Emotional response Helplessness Reduction in suffering Dealing with suffering Current death process	“The pain for everyone involved watching someone suffer while having no quality of life” (Pharmacist 69) “I see people at the end of life, stuck in bodies that no longer function and these people are often in pain” (Pharmacist 117) “Seeing patients and knowing what they must go through” (Pharmacist 167)
Autonomy	Choice Freedom to decide Personal experience with friends or family Emotional responses	“Speaking to a specialist palliative care pharmacist leader, and an ethicist as part of the information gathering helped me understand my own “philosophies” and personal feelings on assisted dying”. (Pharmacist 8) “I still believe in and support the hospice aim to improve quality of life until the end, but my some of my experiences there and the people I encountered have lead me to feel quite strongly that individuals should have the right to end their suffering if they choose” (Pharmacist 188) “Working in the environment I do—I find that hope drives the decisions we make to fight help people live. I struggle to reconcile my fight for hope with assisted dying” (Pharmacist 325)

**Table 3:** Main concerns around legalising medically assisted dying in New Zealand.

Emerging Theme	Subtheme	Quote
Legality	Criminality Legalities Enforcement Historical Precedence Exemptions Interpretation disagreement Accountability	“The legislation will be under great pressure to extend it to other inappropriate groups” (Pharmacist 40) “The regulations around it have to be tight enough that people won’t be able to do it on a whim, along with children who can’t understand the decision fully or people who will try to misuse it” (Pharmacist 228) “The current legislation is very loose and extremely short for something as important as the ending of a person’s life, especially when considering other medical legislation within NZ law and comparisons to legislation in other countries on assisted dying. It does not provide safeguards to the widening of criteria” (Pharmacist 296)
Personal Bias	Objective bias Personal opinions Conscious bias	“I’m concerned about supplying the medicine myself, I support the bill but don’t want to be involved in supplying the medicine” (Pharmacist 26) “I do not believe providing this tool under a legislation as national ‘act’ is in any way promoting a human beings right of life” (Pharmacist 43) “Personally I think it’s such a burden to place on medical professionals. This includes pharmacists- who may have to dispense medication that is intended for this use” (Pharmacist 156)
Palliation	Service provision Lack of service offer Role of service Alternative service provision	“Ensuring that the person has had good, appropriate access to palliative care and mental health services BEFORE the decision to take the medically assisted dying route” (Pharmacist 8) “Less money could potentially be spent on hospice and end-of-life care leading to people who do NOT choose medically assisted dying being left with fewer options for treatment and care” (Pharmacist 148) “That palliative care will continue to be underfunded leaving people without access to adequate palliative are and then they feel like they don’t have any other option but to seek medically assisted dying, when this option could be avoided” (Pharmacist 224)
Stigma	Personal Professional Pharmacist Peers Fear Discrimination	“Patients and providers being publicly persecuted for using or providing the service” (Pharmacist 15) “Pressure from people who don’t think similarly—as always, there are always those who will spread misinformation about the Act and its consequences” (Pharmacist 114) “Potential to put the profession in a ‘tough place’—should it get to the stage where people are collecting the drugs needed to carry out the medically assisted suicide from the pharmacy, that would be extremely distressing…. this goes against the core principle that a healthcare professional should be able to practise according to their own beliefs and values” (Pharmacist 179)
Vulnerability	Professional ethics Power of attorney Disability Access Rural care Inappropriate influence Coercion Timing to access	“I am concerned that this will become an expectation, and that those with terminal illness will feel obliged to take this route. I am also concerned that people will feel coerced into this” (Pharmacist 31) “The ‘right’ of an individual to choose to die may become a ‘duty” to die for reasons apart from what it is originally outlined, such as pressure from burdened family members, financial hardships, emotional distress or psychological conditions that may hinder their abilities to make the right and honest decision” (Pharmacist 43) “Detecting coercion from the patient is difficult—we do not know what happens behind close doors and how much pressure the patient may have been given to influence their decision to take this choice. …Several long-term conditions that cannot be cured, such as Multiple Sclerosis, that cause disability are also terminal illnesses hence will meet the criteria” (Pharmacist 221)

**Table 4:** Services participants would be willing to provide to support the provision of legally assisted dying.

Emerging Theme	Subtheme	Quote
Legal	Referral to medical practitioner Scope of Practice Obtain consent Obedience to Legislation	“I would have no concerns discussing the Act and process—as described within the permissions of the Act” (Pharmacist 8) “Whatever that is relevant to our scope of practice” (Pharmacist 89) “Services consistent with the role of a pharmacist” (Pharmacist 265)
Professionalism	Counselling and support Whanau support Provision of some service Service awareness Information queries Multi-disciplinary team meetings	“Counselling and support of patient” (Pharmacist 4) “Supportive services to outline what other services are available” (Pharmacist 112) “Whatever is asked of me by any GP I work with who may provide the service” (Pharmacist 114)
Service/Access	Dispensing/preparation of medicine Provision of medication Procedural information Delivery of medications Provision of leaflets Procurement of medicine Medication review	“I would be willing to help supply or in a collaborative setting provide support through the process or administering of such a service” (Pharmacist 35) “Procurement and preparation of medicines involved” (Pharmacist 57) “Dispensing prescriptions; advice on use of end of life medicines” (Pharmacist 182)
Education	Education Upskilling	” Education re medication provision and admin” (Pharmacist 13) “Advice on how the medicine works, how to take it, how quickly it works. If they were unsure then advice about other pain relieving or other treatments that may ease their suffering a bit if pain was a problem (so if their medicine management was suboptimal)” (Pharmacist 54) “Education of people or medical staff as appropriate” (Pharmacist 156)

## Data Availability

The data presented in this study are available on request from the corresponding author. The data are not publicly available due to ethical and privacy restrictions.
